# An Unusual Dysphagia for Solids in a 17-Year-Old Girl Due To a Lusoria Artery: A Case Report and Review of the Literature

**DOI:** 10.3390/ijerph17103581

**Published:** 2020-05-20

**Authors:** Umberto Fanelli, Rosanna Iannarella, Aniello Meoli, Pierpacifico Gismondi, Simone Cella, Francesca Vincenzi, Susanna Esposito

**Affiliations:** 1 Paediatric Clinic, Pietro Barilla Children’s Hospital, Department of Medicine and Surgery, University of Parma, 43121 Parma, Italy; umbertaker@msn.com (U.F.); rosanna.iannarella@hotmail.it (R.I.); aniello.meoli@gmail.com (A.M.); pgismondi@ao.pr.it (P.G.); 2 Unit of Paediatric Radiology, Pietro Barilla Children’s Hospital, Department of Medicine and Surgery, University of Parma, 43121 Parma, Italy; cella@ao.pr.it; 3 Unit of Gastroenterology and Digestive Endoscopy, Department of Medicine and Surgery, University of Parma, 43121 Parma, Italy; VincenziF@ao.pr.it

**Keywords:** computed tomography angiography dysphagia, lusoria artery, nutrition, oesophagogastroduodenoscopy

## Abstract

*Background:* Dysphagia is a condition that can have many underlying causes, often different between adults and children and its early diagnosis is crucial especially during childhood and adolescence, given the importance of proper nutritional intake to ensure adequate growth and development. *Case report:* We described the case of a 17-year-old girl reporting dysphagia for solids for approximately one month. No symptoms were previously referred. Oesophagogastroduodenoscopy was performed, detecting an image of ab extrinseco compression at the level of the mid-cervical oesophagus. An upper gastrointestinal tract radiography confirmed an oesophageal impression above the arch of the aorta suggestive of vascular abnormality. Computed tomography angiography and three-dimensional reconstruction techniques showed the presence of a lusoria artery that originated from the medial margin of the descending aorta and crossed the trachea and oesophagus posteriorly to the distal third. The lusoria artery was transected via a left thoracotomy and re-implanted into the right common carotid artery with complete symptom resolution. *Conclusions:* Dysphagia lusoria is an impairment of swallowing due to compression from an aberrant right subclavian artery. The diagnosis is always difficult, as the symptoms are often nonspecific. It is imperative to accurately identify and properly manage dysphagia in pediatric age and this is only possible with an anamnestic, clinical and instrumental process that takes into account an adequate differential diagnosis.

## 1. Introduction

Dysphagia is defined as any disruption to the swallow sequence that results in the compromise of the safety, efficacy or adequacy of nutritional intake [1]. The research suggests that about 1% of otherwise healthy children and adolescents may have difficulties in swallowing [2,3] and given the importance of a proper nutritional intake during childhood and adolescence to ensure adequate growth and development (including developmental delays relating to feeding and aversion), early diagnosis and intervention by a multidisciplinary team is essential [4]. Unfortunately, feeding/eating disorders and dysphagia are not always distinguished from each other. Table 1 summarizes the prevalence of dysphagia according to underlying risk factors in pediatric age [5]. The underlying causes of dysphagia may be different in pediatric age and adulthood [6]. 

The assessment of a child or adolescent presenting symptoms related to a swallow dysfunction primarily consists of an effective medical history and objective examination and continues with a series of investigations including the clinical feeding evaluation, the video fluoroscopic swallow study, upper gastrointestinal radiological series and endoscopic evaluations (including the fiberoptic endoscopic evaluation of swallowing). In terms of treatment, the pediatric patient with dysphagia benefits from a specialized multidisciplinary approach that includes pediatricians, neurologists, otolaryngologists, pulmonologists, gastroenterologists, dietitians, occupational therapists and speech-language pathologists [7]. A surgical approach can instead be considered in the pediatric field when an anatomical anomaly is identified as the cause of dysphagia. We described the case of a 17-year-old girl reporting dysphagia for solids for approximately one month due to a lusoria artery.

## 2. Case Report

A 17-year-old girl was admitted to the Pediatric Clinic of our Children’s Hospital for the appearance of sudden dysphagia for solids for about a month. No symptoms were previously referred. In previous medical history, only allergy to dust mites and sideropenic anemia in supplementary therapy with iron was reported. Thyroid ultrasound as well as screening for celiac disease and thyroid function were performed with normal results. Positivity for *Helicobacter pylori* infection was also reported.

Upon arrival, the girl was in good general condition. In the physical examination, normal chest and abdominal objectivity was found. Due to the difficulty in eating, she was on a diet of blended and homogenized foods. The haematochemical tests confirmed sideropenic anemia (Hb 8.5 g/dL, MCV 75.5 fL MCH 22.4 pg MCHC 29.7 g/dL), probably due to a menorrhagia reported in the previous three months.

**Figure 1:**
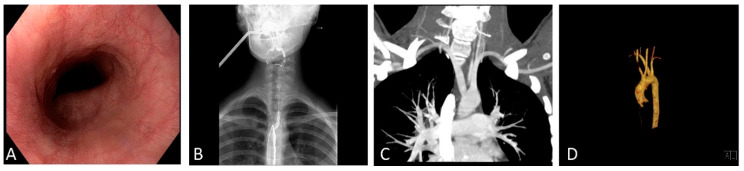
(**A**) Oesophagogastroduodenoscopy showing an image of an ab extrinseco compression of the mid-cervical oesophagus. (**B**) Upper gastrointestinal tract radiography confirming an oesophageal impression above the arch of the aorta. (**C**) Computed tomography angiography and (**D**) three-dimensional visualization, which detect the presence of a lusoria artery originating from the medial margin of the descending aorta and crossing the trachea and oesophagus posteriorly to the distal third, resulting in a tortuous appearance.

According to the symptomatology, an oesophagogastroduodenoscopy was performed, detecting an image from ab extrinsic compression at the level of the middle-cervical oesophagus, apparently pulsating (Figure 1A). At the same time, a slight gastritis of the antrum was reported. The culture test for *H. pylori* on biopsies was negative.

Based on the findings of the endoscopic examination, an upper gastrointestinal tract radiography was initially performed, confirming an oesophageal impression above the arch of the aorta (Figure 1B). Subsequently, suspecting a vascular anomaly, a computed tomography (CT) angiography was requested, showing the presence of a lusoria artery that originated from the medial margin of the descending aorta and crossed through the trachea and oesophagus posteriorly to the distal third, resulting in a tortuous appearance (Figure 1C).

Following a multidisciplinary evaluation, in view of the worsening symptomatology, the patient was transferred to cardiac surgery to assess the possibility of a surgical approach. After a surgical evaluation, a reimplantation of the right carotid subclavian artery was first performed. Imaging tests (CT and upper gastrointestinal tract radiography) performed in the following days showed an oesophageal compression of the arterial stump. A second operation was then performed with a subclavian lusoria artery stump section. The surgery was well tolerated. In the immediate post-operative period, a rapidly resolved Claude Bernard Horner syndrome of the right eye was reported. The patient resumed gradual feeding and was discharged after a month in good general condition. After two further months, she was currently asymptomatic and reported no dysphagia.

## 3. Discussion

Although digestive disorders are the most common causes of dysphagia, there are other less frequent diseases that must be excluded from the diagnostic process. This group includes disorders caused by vascular compression of the oesophagus on the mediastinum.

Dysphagia lusoria is defined as a swallowing disorder secondary to the extrinsic compression of the oesophagus by vascular structures. Abnormalities of any mediastinal vessel can potentially cause this pathology, but the most common one described in the literature is represented by an aberrant right subclavian artery (ARSA). This is related to the fact that it is the most common congenital alteration of the aortic arch [8]. 

The most frequent point of origin of lusoria artery is the so-called “Kommerell’s diverticulum”, an aortic diverticulum. Normally, during the embryonic development a double aortic system develops which requires the subsequent obliteration of different components to ensure normal vascular anatomy. If for some reason this process is incomplete or inadequate, it will produce one of the alterations of the large vessels. Among the many classification systems, the most common one divides them into five main groups [9]: (1) a double aortic arch; (2) a right aortic arch with a left arteriosum ligament or persistent duct; (3) an aberrant subclavian artery; (4) a left aberrant pulmonary artery; and (5) alterations of the unnamed artery.

ARSA has an estimated prevalence of 0.4%–2% in the general population and in most cases is asymptomatic. Children may present with recurrent lung infections or stridor while adults more often present with dysphagia lusoria [10]. The lusoria artery follows a retroesophageal course in 80% of cases, passes between the oesophagus and trachea in 15% and is anterior to the trachea or main bronchus in 5% [11]. From a diagnostic point of view, the progress in imaging has made angiographic studies based on CT scans the best in diagnostics, considering their availability and non-invasiveness. With the development of multi-detector TC and 3D reconstructions it is also much easier to analyze the anatomical characteristics of the aortic arch [12], as in our patient (Figure 1D). Oesophagogastroduodenoscopy can instead visualize any mucosal alterations or extrinsic compressions.

With regard to treatment, in moderate cases, lifestyle changes and education on eating behavior can substantially improve the clinical condition of the patient. The surgical approach does not yet have clear indications and its use remains controversial: it depends on the anatomical relief, urgency and experience of the surgeon himself [13]. There are many approaches described in the literature but the main objective remains to remove the aberrant vessel and reconstruct the vascular system in a functional way. Clearly, the risk/benefit ratio of this type of intervention must always be considered given the mortality and morbidity rates [14]. In the pediatric field, a two-site approach seems to be less invasive and more tolerated than the others, often managing to eliminate the symptoms and restore antegrade blood flow to the right arm [15].

The diagnosis of dysphagia lusoria is complex because the symptoms are often non-specific; at the same time the oesophagogastroduodenoscopy is negative in 50% of cases [16]. Although symptoms may appear at any age, they generally tend to occur around the end of the fifth decade of life; it is suspected that this is due to changes in the oesophagus and blood vessels related to age and due to the decrease in the elasticity and stretchability of these anatomical components. In the case of our patient, it is possible that the development of hyperdynamic circulation, as a result of an anemia condition, triggered the development of symptomatology.

## 4. Conclusions

It is imperative to accurately identify and properly manage dysphagia in pediatric age, even more readily than in adults because children and adolescents are subject to a rapid development of the body and even minor swallowing alterations could interrupt growth and cause long-term sequelae. This is only possible with an anamnestic, clinical and instrumental process that takes into account an adequate differential diagnosis.

## Figures and Tables

**Table 1:** Prevalence of dysphagia according to the underlying risk factors in pediatric age.

Risk Factor	Prevalence
None	0.9%
Developmental disorder	30%–80%
Cerebral palsy	19.2%–99.0%
Suspected gastrointestinal disorders or eating disorders	1.5%–13.8%
Craniofacial disorder	33%–83%

Adapted from [5].

## References

[1] Dodrill P., Gosa M.M. (2015). Pediatric dysphagia: Physiology, assessment, and management. Ann. Nutr. Metab..

[2] Bhattacharyya N. (2015). The prevalence of pediatric voice and swallowing problems in the United States. Laryngoscope.

[3] Raol N., Schrepfer T., Hartnick C. (2018). Aspiration and dysphagia in the neonatal patient. Clin. Perinatol..

[4] Kakodkar K., Schroeder J.W. (2013). Pediatric dysphagia. Pediatr. Clin. North Am..

[5] American Speech Language Hearing Association Pediatric Dysphagia. https://www.asha.org/PRPSpecificTopic.aspx?folderid=8589934965&section=Incidence_and_Prevalence.

[6] Newman L.A., Keckley C., Petersen M.C., Hamner A. (2001). Swallowing function and medical diagnoses in infants suspected of dysphagia. Pediatrics.

[7] Borowitz K.C., Borowitz S.M. (2018). Feeding problems in infants and children: Assessment and etiology. Pediatr. Clin. North Am..

[8] de Araújo G., Junqueira Bizzi J.W., Muller J., Cavazzola L.T. (2015). “Dysphagia lusoria”—Right subclavian retroesophageal artery causing intermitent esophageal compression and eventual dysphagia—A case report and literature review. Int. J. Surg. Case Rep..

[9] Ozturk E., Karaman B., Sonmez G., Sildiroglu H.O., Mutlu H., Velioglu M. (2006). Right aortic arch with left subclavian artery arising from Kommerell’s diverticulum. Eur. J. Radiol. Extra.

[10] Klin B., Tauber Z., Peer A., Broide E., Vinograd I., Bass A. (1996). Dysphagia lusoria in children. Eur. J. Vasc. Endovasc. Surg..

[11] Stone W.M., Ricotta J.J., Fowl R.J. (2011). Contemporary management of aberrant right subclavian arteries. Ann. Vasc. Surg..

[12] Keum B., Kim Y.S., Jeen Y.T., Chun H.J., Um S.H., Kim C.D., Ryu H.S., Hyun J.H. (2006). Dysphagia lusoria assessed by 3-dimensional TC. Gastrointest. Endosc..

[13] Álvarez J.R., Quiroga S.J., Nazar A.B., Comendador M.J., Carro G.J. (2008). Aberrant right subclavian artery and calcifi ed aneurysm of Kommerell’s diverticulum: An alternative approach. J. Cardiothorac. Surg..

[14] Smith J.M., Reul G.J., Wukasch D.C., Cooley D.A. (1979). Retroesophageal subclavian arteries: Surgical management of symptomatic children. Cardiovasc. Dis..

[15] Nelson J.S., Hurtado C.G., Wearden P.D. (2020). Surgery for Dysphagia Lusoria in Children. Ann. Thorac. Surg..

[16] Janssen M., Baggen M.G., Veen H.F., Smout A.J., Bekkers J.A., Jonkman J.G., Ouwendijk R.J. (2000). Dysphagia lusoria: Clinical aspects, manometric findings, diagnosis, and therapy. Am. J. Gastroenterol..

